# The Development of AGHE as a Force in Gerontology Education

**DOI:** 10.1093/geroni/igaa057.1810

**Published:** 2020-12-16

**Authors:** Harvey Sterns

**Affiliations:** The University of Akron, Akron, Ohio, United States

## Abstract

This presentation will focus on the history and development of AGHE beginning in 1972. The issues and concerns of early leaders will be reviewed base on articles, books and early publications of the association. There are core issues of gerontology education that continue to be challenges to the present. Significant contributions to these early years will be highlighted. Part of a symposium sponsored by the Geriatric Education Interest Group.

